# Supplementary material for: Cytoplasmic localization of the mRNA encoding actin regulator, Serendipity-α, promotes adherens junction assembly and nuclear repositioning
Source: bioRxiv. 2025 Jul 31:2025.07.29.667534. Preprint. [Version 1] doi: 10.1101/2025.07.29.667534 (PMC12324375; doi:10.1101/2025.07.29.667534)
Supplement: Supplement 5 [file media-5.pdf]

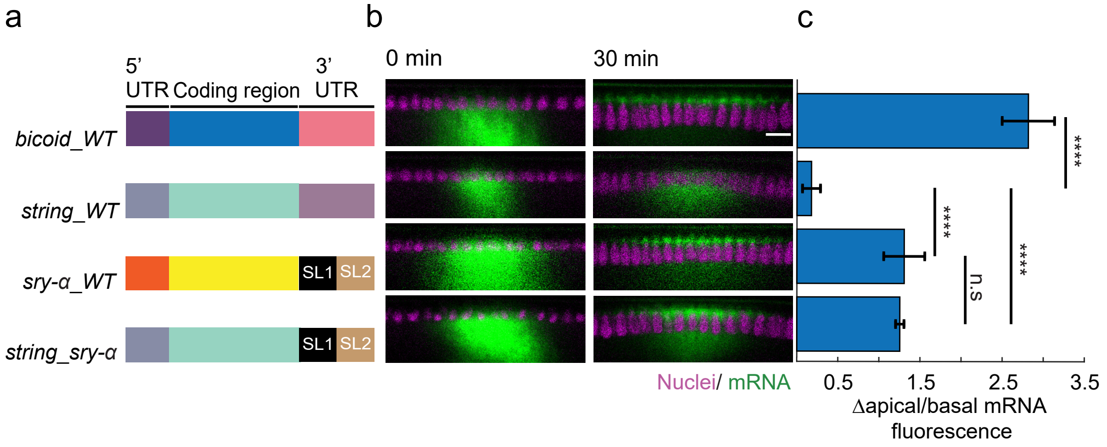

a
5' UTR
Coding region
3' UTR
bicoid_WT
string_WT
sry-α_WT
string_sry-α
SL1
SL2
b
0 min
30 min
Nuclei/ mRNA
c
****
****
n.s
****
0.5
1.5
2.5
3.5
Δapical/basal mRNA fluorescence

Extended Data Fig. 1 | Related to Fig. 3e-g

a, Schematic shows construct design for *in vitro* transcribed mRNAs for positive control (*bicoid_WT*), negative control (*string_WT*) and *sry-α* experimentals (*sry-α_WT*, *string_sry-α*; WT, wild-type; SL, stem loop). **b**, From time-lapse imaging, cross-sections show injected mRNA (green) and nuclei (Histone-mCherry, magenta). Identity of the mRNA is indicated by aligned construct in (**a**). Imaging started immediately after injection and proceeded for 30 minutes (0 min and 30 min, respectively). Scale bar = 10 μm. **c**, Change in apical to basal fluorescence from 0 to 30 minutes for the mRNA indicated by aligned construct in (**a**). Bars indicate mean ± s.e.m. (n = 5 embryos per construct); $p > 0.05$, not significant (n.s.); ****$p < 0.00005$, one-way ANOVA.

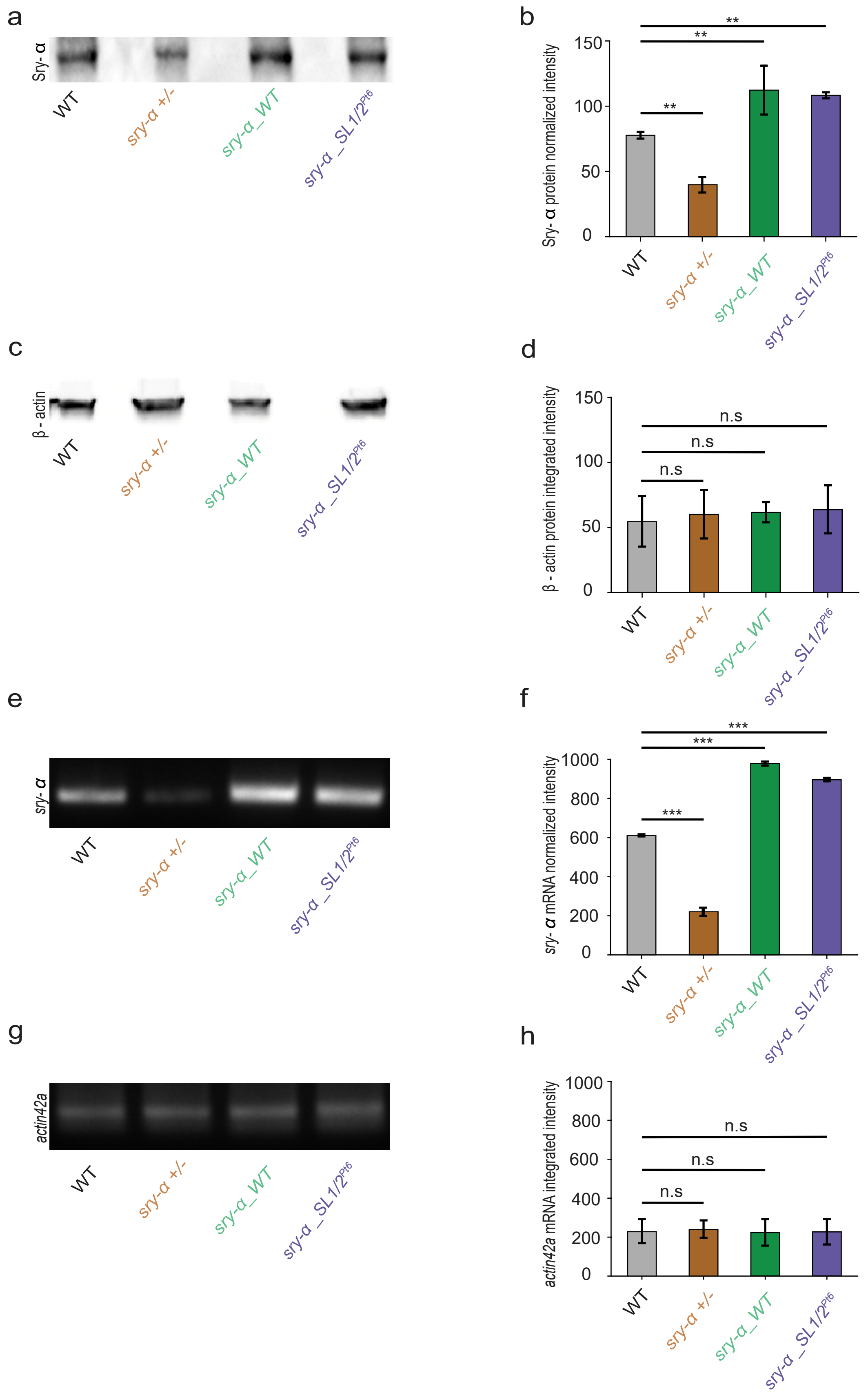

a
Sry- α
WT
sry-α +/-
sry-α_WT
sry-α _SL1/2Pt6
b
Sry- α protein normalized intensity
150
100
50
0
**
**
**
c
β - actin
d
β - actin protein integrated intensity
n.s
n.s
n.s
e
sry- α
f
sry- α mRNA normalized intensity
1000
800
600
400
200
0
***
***
***
g
actin42a
h
actin42a mRNA integrated intensity
n.s
n.s
n.s

Extended Data Fig. 2 | Related to Fig. 3h, i

a,**c**, Immunoblots show Sry-α and β-actin bands, respectively, for protein lysates from embryos from indicated stocks. **b**,**d**, Quantification of Sry-α and β-actin protein levels, respectively, as determined by densitometry. **e**,**g**, Ethidium bromide stained agarose gels show reverse transcription-PCR products for *sry-α* and *actin42a*, respectively, following mRNA extraction from embryos from indicated stocks. **f**,**h**, Quantification of *sry-α* and *actin42a* PCR product levels, respectively, as determined by densitometry. PCR product levels are considered a proxy for mRNA levels in embryos.

a-**h**, Stocks are: OreR (wild-type, WT); *sry-α - /TM3Sb, hb::LacZ (sry-α +/-)*; *sry-α_WT; sry-α_SL1/2*^{Pt6}.

b,**d**,**f**,**h**, Bars indicate mean ± s.e.m. (n = 3 biological replicates); $p > 0.05$, not significant (n.s.); **$p < 0.005$, ***$p < 0.0005$, ****$p < 0.00005$, Students t-test.

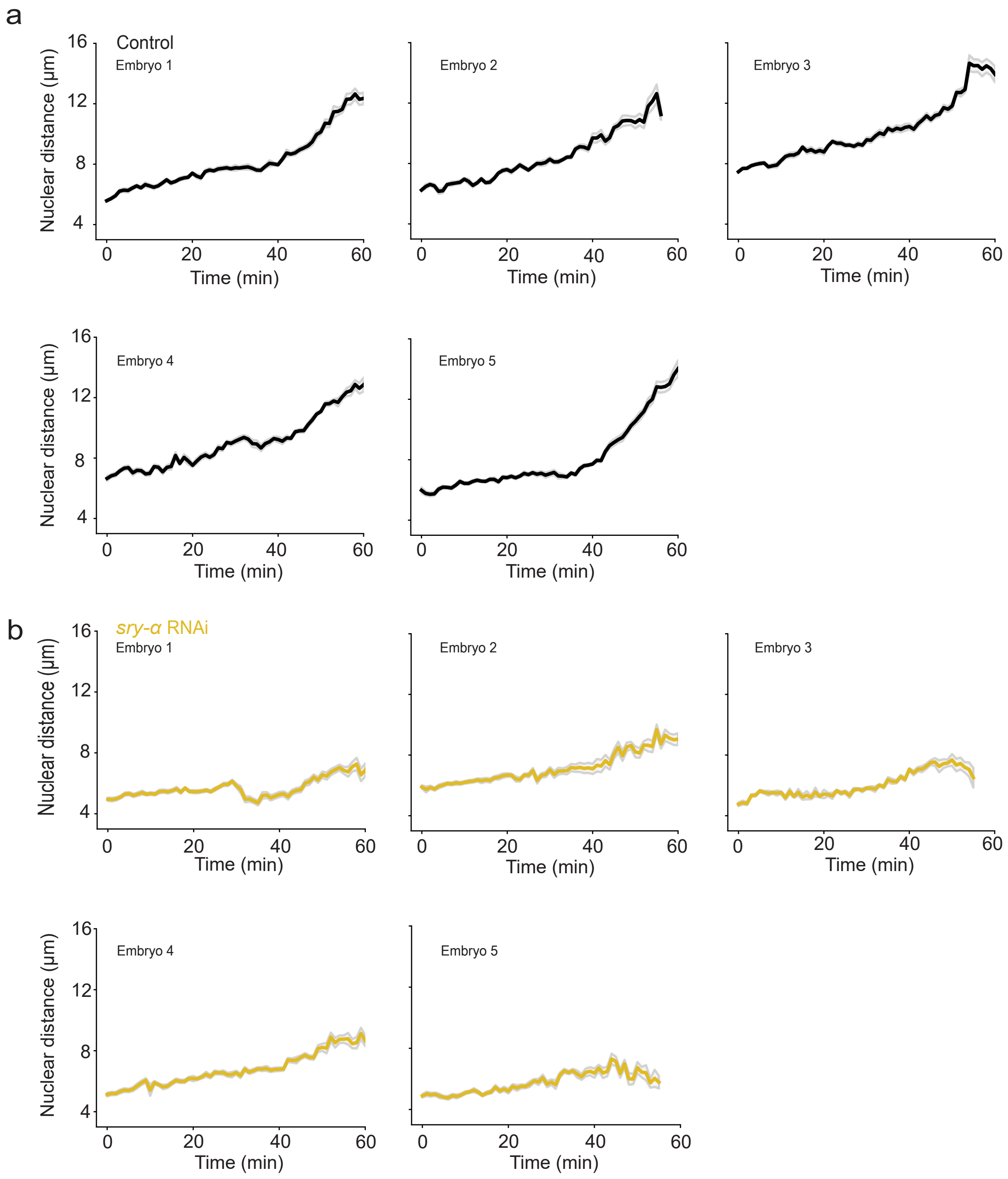

a
Control
Embryo 1
Embryo 2
Embryo 3
Embryo 4
Embryo 5
Nuclear distance (µm)
Time (min)
b
sry-α RNAi
Embryo 1
Embryo 2
Embryo 3
Embryo 4
Embryo 5
Nuclear distance (µm)
Time (min)

Extended Data Fig. 3 | Related to Fig. 4

a,**b**, Individual embryo, raw nuclear distances for buffer (Control, black, **a**) and *sry-α* dsRNA (*sry-α* RNAi, gold, **b**) injected embryos over the course of cellularization (15 nuclei followed per embryo; mean ± s.e.m. demarcated in gray).

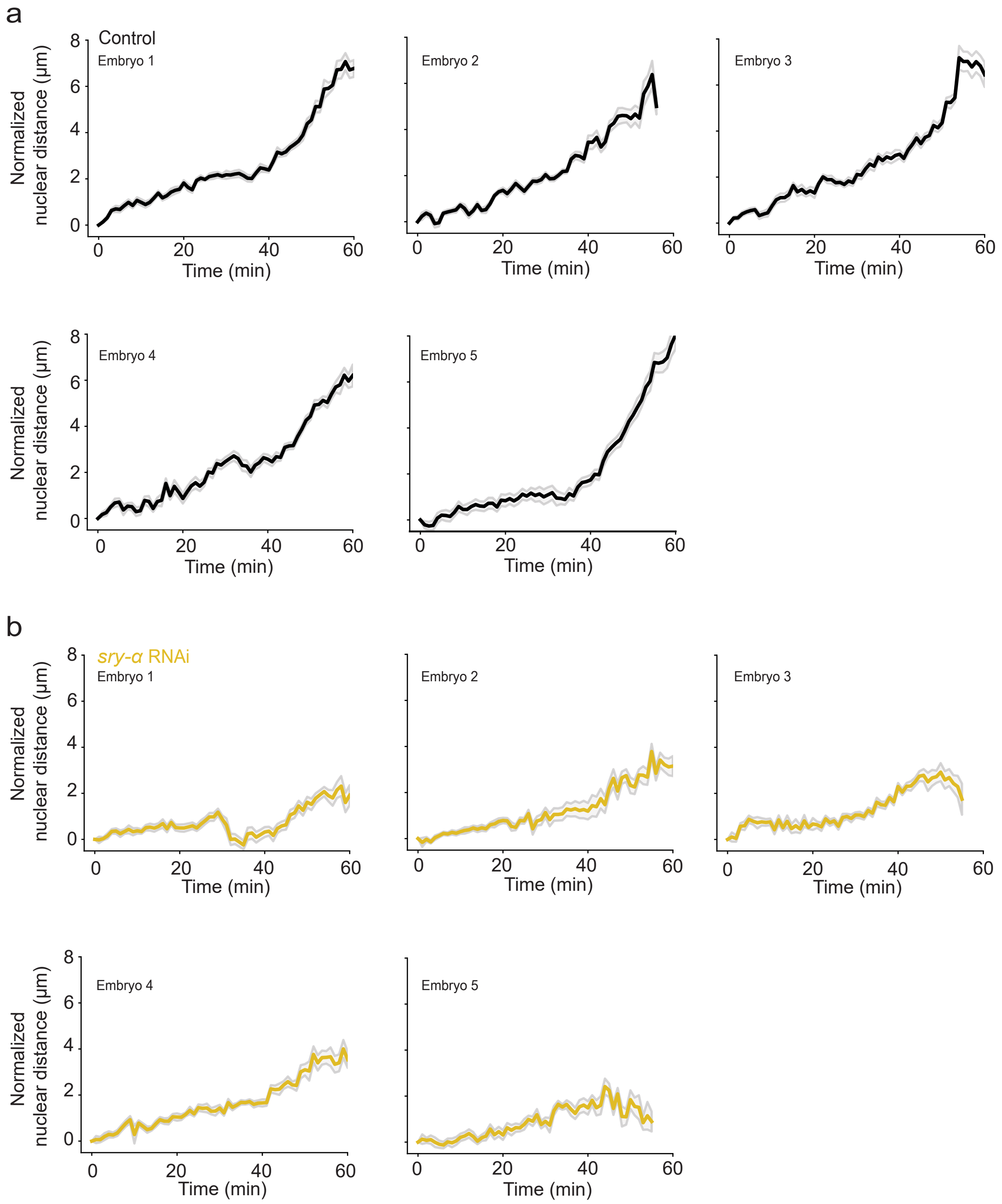
a
Control
Embryo 1
Embryo 2
Embryo 3
Embryo 4
Embryo 5
Normalized nuclear distance (μm)
Time (min)
0
20
40
60
2
4
6
8
b
sry-α RNAi
Embryo 1
Embryo 2
Embryo 3
Embryo 4
Embryo 5
Normalized nuclear distance (μm)
Time (min)

Extended Data Fig. 4 | Related to Fig. 4

a,**b**, Individual embryo, normalized nuclear distances for buffer (Control, black, **a**) and *sry-α* dsRNA (*sry-α* RNAi, gold, **b**) injected embryos over the course of cellularization (15 nuclei followed per embryo; mean ± s.e.m. demarcated in gray).

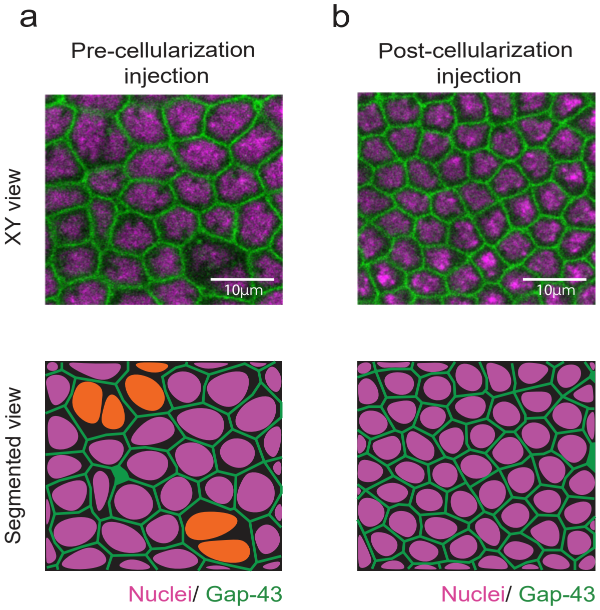
a
b
Pre-cellularization injection
Post-cellularization injection
XY view
10μm
10μm
Segmented view
Nuclei/ Gap-43
Nuclei/ Gap-43

Extended Data Fig. 5 | Related to Fig. 6

a,**b**, From time-lapse imaging, single plane confocal XY views show cellularization furrows (Gap43-GFP / plasma membrane, green) ingressing between nuclei (Histone-mCherry, magenta) in *sry-α* morpholino injected embryos. Embryos were either injected prior to cellularization (**a**) or just after cellularization onset (**b**; n = 5 embryos per treatment). Bottom row shows segmented views of the corresponding XY views with nuclei highlighted (orange, **a**) in multinucleated cells where furrows regressed. Images collected from embryos at late cellularization, with furrow lengths > 5 μm. Scale bars = 10 μm.

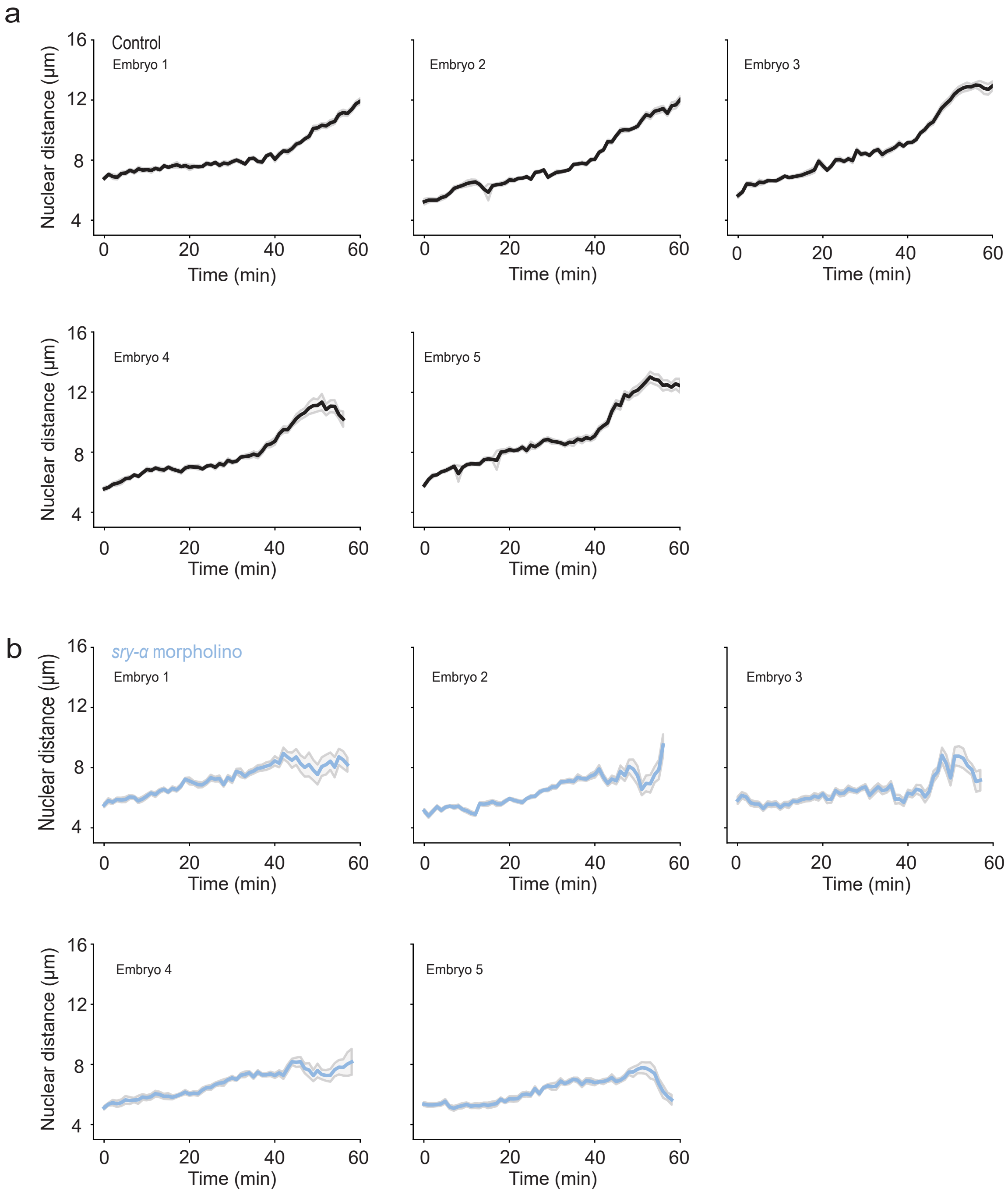

a
Control
Embryo 1
Embryo 2
Embryo 3
Embryo 4
Embryo 5
Nuclear distance (µm)
Time (min)
b
sry-α morpholino
Embryo 1
Embryo 2
Embryo 3
Embryo 4
Embryo 5
Nuclear distance (µm)
Time (min)

Extended Data Fig. 6 | Related to Fig. 6

a,**b**, Individual embryo, raw nuclear distances for control morpholino (Control, black, **a**) and *sry-α* morpholino (*sry-α* morpholino, blue, **b**) injected embryos over the course of cellularization (15 nuclei followed per embryo; mean ± s.e.m. demarcated in gray).

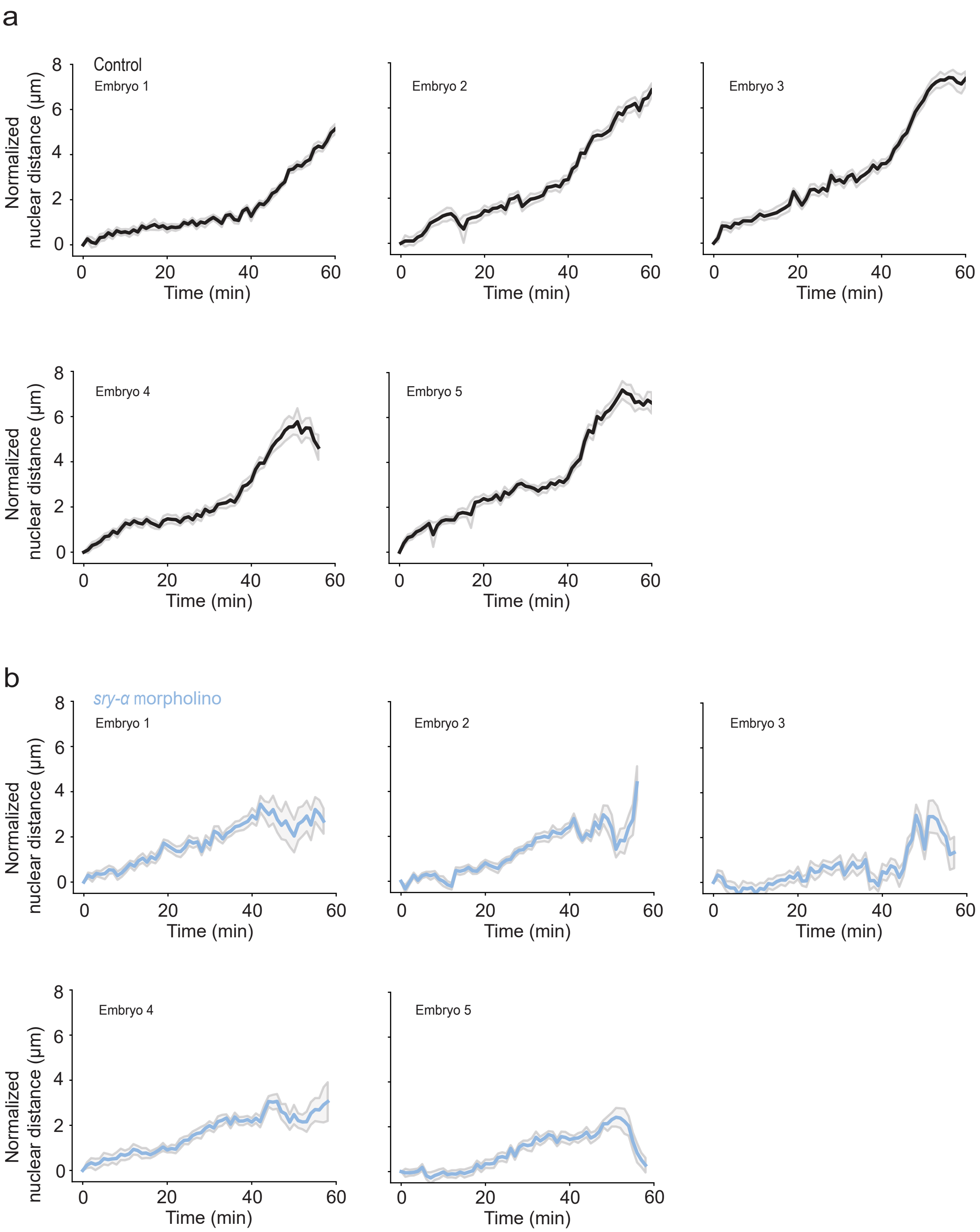
a
Control
Embryo 1
Embryo 2
Embryo 3
Embryo 4
Embryo 5
Normalized nuclear distance (μm)
Time (min)
0
2
4
6
8
0
20
40
60
b
sry-α morpholino
Embryo 1
Embryo 2
Embryo 3
Embryo 4
Embryo 5
Normalized nuclear distance (μm)
Time (min)

Extended Data Fig. 7 | Related to Fig. 6

a,**b**, Individual embryo, normalized nuclear distances for control morpholino (Control, black, **a**) and *sry-α* morpholino (*sry-α* morpholino, blue, **b**) injected embryos over the course of cellularization (15 nuclei followed per embryo; mean $\pm$ s.e.m. demarcated in gray).

Extended Data Video 1 (Related to Fig. 4): Time lapse movie showing nuclear repositioning over the course of cellularization in a buffer-injected embryo (Control). Time is shown in minutes. 1 minute indicates the beginning of cellularization. Nuclei (magenta) elongate and move to a more basal position as cellularization proceeds. The vitelline membrane (orange line) serves as a marker for the embryo surface. Arrow shows relative movement of a single nucleus.

Extended Data Video 2 (Related to Fig. 4): Time lapse movie showing nuclear repositioning over the course of cellularization in a *sry-α* dsRNA-injected embryo (*sry-α* RNAi). Time is shown in minutes. 1 minute indicates the beginning of cellularization. Nuclei (magenta) elongate but fail to move to a more basal position as cellularization proceeds. Some nuclei move basally but reverse direction and "pop-up" to a very apical position. Some nuclei also tilt such that their long axis no longer remains at the normal 90° orientation to the cell surface. The vitelline membrane (orange line) serves as a marker for the embryo surface. Arrow shows relative movement of a single nucleus. Asterisks mark nuclei that "pop-up".

Extended Data Video 3 (Related to Fig. 6): Time lapse movie showing nuclear repositioning over the course of cellularization in a control morpholino injected embryo (Control). Time is shown in minutes. 1 minute indicates the beginning of cellularization. Nuclei (magenta) elongate and move to a more basal position as cellularization proceeds. The vitelline membrane (orange line) serves as a marker for the embryo surface. Arrow shows relative movement of a single nucleus.

Extended Data Video 4 (Related to Fig. 6): Time lapse movie showing nuclear repositioning over the course of cellularization in a *sry-α* morpholino injected embryo (*sry-α* morpholino). Time is shown in minutes. 1 minute indicates the beginning of cellularization. Nuclei (magenta) elongate but fail to move to a more basal position as cellularization proceeds. Some nuclei move basally but reverse direction and “pop-up” to a very apical position. The vitelline membrane (orange line) serves as a marker for the embryo surface. Arrow shows relative movement of a single nucleus. Asterisks mark nuclei that “pop-up”.
